# A case report of contrast‐enhanced harmonic ultrasonography for the diagnosis of an esophageal duplication cyst

**DOI:** 10.1002/deo2.218

**Published:** 2023-03-17

**Authors:** Kazunori Adachi, Takashi Tashiro, Makiko Kato, Takanori Ito, Aya Kato, Masaaki Takayama, Shunsuke Kato, Satoshi Ono, Hisako Yoshimine, Akira Koshino, Kazuhiro Nagao, Yuji Kobayashi, Masahide Ebi, Naotaka Ogasawara, Makoto Sasaki, Kunio Kasugai

**Affiliations:** ^1^ Department of Gastroenterology Aichi Medical University Hospital Aichi Japan; ^2^ Department of Surgical Pathology Aichi Medical University Hospital Aichi Japan

**Keywords:** endoscopic ultrasonography, fine‐needle aspiration, submucosal tumor, esophageal cyst, thoracoscopic surgery

## Abstract

A 54‐year‐old man was referred to our hospital because of a suspected esophageal submucosal tumor on upper gastrointestinal radiography. Contrast‐enhanced computed tomography showed a 52 mm homogeneous mass attached to the lower thoracic esophagus. Esophagogastroduodenoscopy revealed a 50 mm submucosal tumor in the lower esophagus, and endoscopic ultrasonography (EUS) showed a continuous hypoechoic lesion in the esophageal muscularis propria. Contrast‐enhanced harmonic EUS revealed a non‐echogenic area. T1 and T2 magnetic resonance imaging revealed a high‐signal lesion. Based on imaging studies, an esophageal duplication cyst was diagnosed. Although asymptomatic, the patient underwent video‐assisted thoracic surgery because of the possibility of rupture and the appearance of symptoms due to a future infection or enlargement, although this was not noted before.

In our case, the esophageal duplication cyst appeared as a hypoechoic mass, requiring differentiation from submucosal tumor other than the cyst. Histologically, the cyst was covered by two layers of muscle covered by the chorioepithelial columnar epithelium. EUS fine‐needle aspiration is effective in diagnosing submucosal tumor but also carries the risk of infection. Contrast‐enhanced ultrasonography was used in this case to observe the interior and reach a preoperative diagnosis. Contrast‐enhanced harmonic EUS appears to be effective in examining the interior of submucosal tumor lesions noninvasively.

## INTRODUCTION

Esophageal duplication cysts are a rare congenital malformation. They are sometimes discovered during childhood and other times in adulthood. They may be found by chance, be asymptomatic, or present with symptoms such as abscess formation or dysphagia.

Magnetic resonance imaging (MRI) and endoscopic ultrasonography (EUS) are often used to diagnose the lesions, but care must be taken when making the diagnosis because the lesion may appear differently, depending on the hemorrhage, protein, or infection inside the cyst. Contrast‐enhanced harmonic EUS (CH‐EUS) is effective in observing the interior of the lesion but has not yet been reported for esophageal duplication cysts.

## CASE REPORT

A 54‐year‐old male was referred to our hospital for a suspected esophageal submucosal tumor on upper gastrointestinal radiography (UGI). Physical and clinical examinations revealed no abnormal findings or subjective symptoms. He had undergone esophagogastroduodenoscopy at another hospital 2 years earlier, but no abnormalities were noted.

**FIGURE 1:**
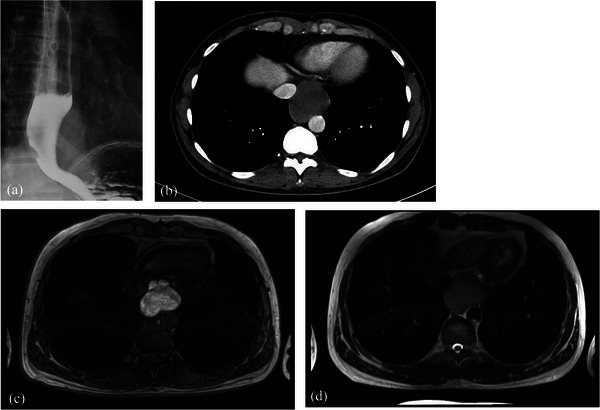
(a) Upper gastrointestinal radiography shows a lesion 50 mm in diameter on the left wall of the lower esophagus. (b) Contrast‐enhanced computed tomography shows a 52 × 44 mm homogeneous mass attached to the lower thoracic esophagus. (c) T1‐weighted magnetic resonance imaging reveals the mass to be multifocal with a high signal. (d) T2‐weighted magnetic resonance imaging reveals a slightly high signal for the mass.

**FIGURE 2:**
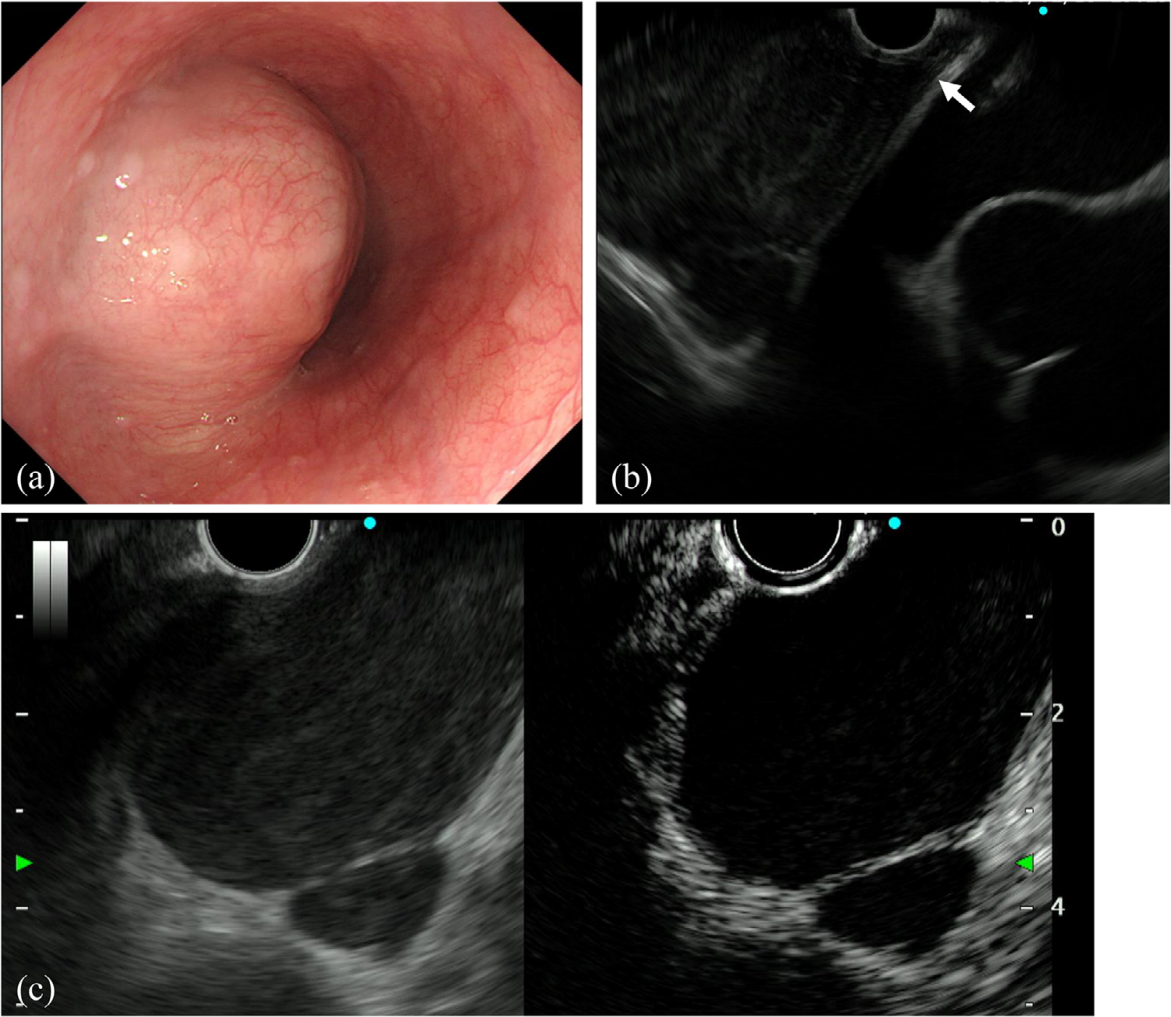
(a) A large mass with a gentle elevation and no epithelial changes causing narrowing of the lower esophageal lumen on esophagogastroduodenoscopy. (b) A hypoechoic lesion contiguous with the intrinsic muscular layer of the esophagus (arrow) is observed on endoscopic ultrasonography (7.5 MHz). (c) Multifocal hypoechoic areas are delineated in the anechoic area with a uniform wall structure on contrast‐enhanced harmonic ultrasonography (7.5 MHz).

UGI revealed a 50 mm large, gently elevated lesion on the left wall of the lower esophagus (Figure 1a). Contrast‐enhanced computed tomography revealed a 52 × 44 mm homogeneous mass attached to the lower thoracic esophagus (Figure 1b). Esophagogastroduodenoscopy revealed a gently sloping mass with stenosis but without epithelial changes (Figure 2a), and EUS revealed a hypoechoic lesion contiguous with the intrinsic muscular layer of the esophagus (Figure 2b). CH‐EUS revealed multifocal anechoic areas with a uniform wall structure (Figure 2c). T1‐weighted MRI revealed the mass to be multifocal with high signal intensity. T2‐weighted MRI revealed a slightly high‐signal intensity of the mass (Figure 1c,d).

An esophageal duplication cyst was diagnosed based on the examination results. Although the cyst was asymptomatic, it had not been noted previously; after informing the patient of the possibility of future growth and the risk of infection, rupture, and impaired passage, the patient requested surgery. Video‐assisted thoracic surgery was performed for the cyst. The lesion appeared to be an esophageal submucosal tumor and was resected along with a portion of the esophageal muscle layer. The missing muscle layers were stitched together.

**FIGURE 3:**
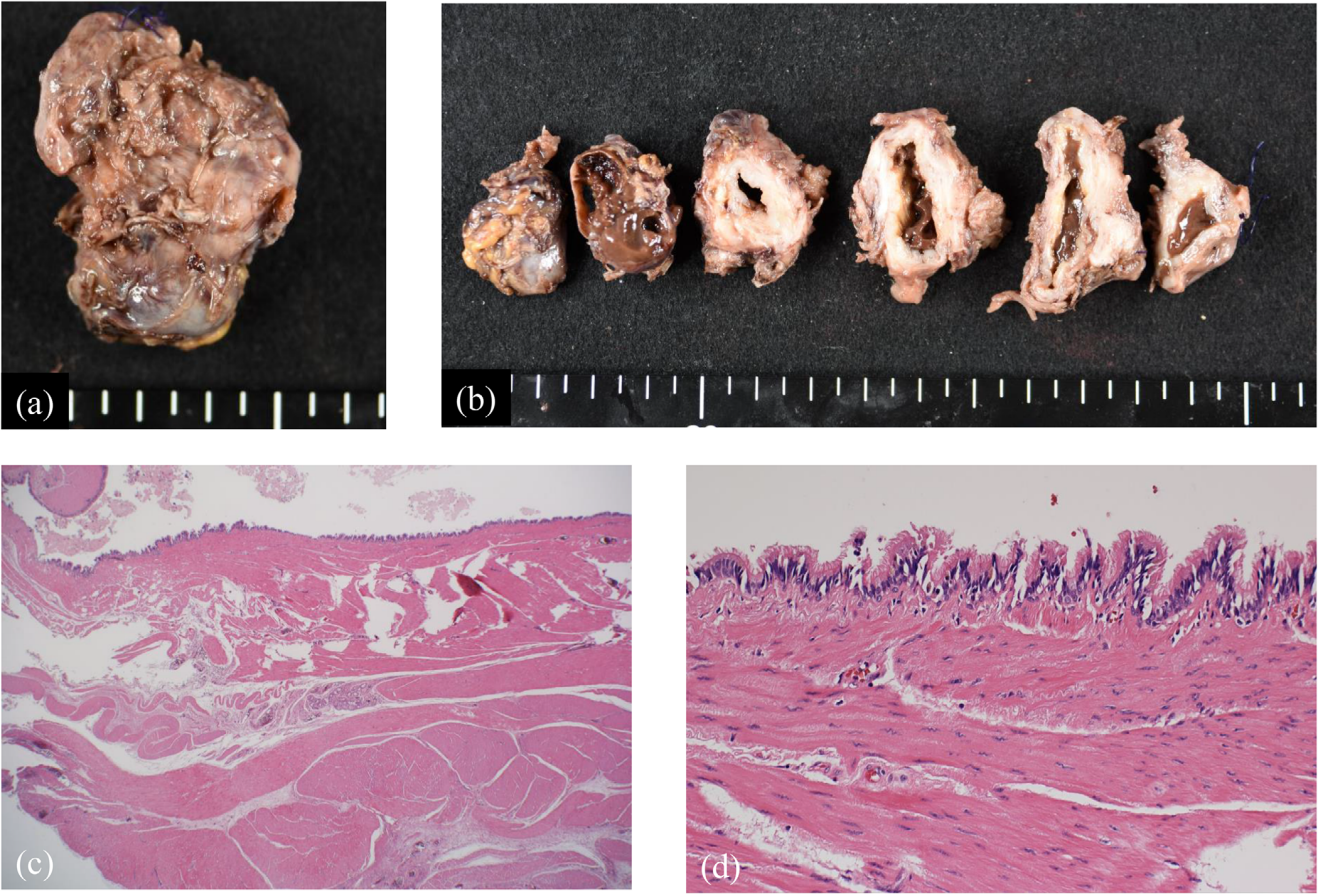
(a) Resected specimen. Its size is 50 × 40 mm. (b) The lesion is filled with a viscous, brownish liquid and appears to be a multifocal cystic lesion with some septate walls. (c) The cyst is covered by two muscle layers (hematoxylin and eosin stain, ×20). (d) The cystic portion of the lesion is covered by chorioepithelial columnar epithelium (hematoxylin and eosin stain, ×200).

The resected specimen was 40 × 50 mm in diameter (Figure 3a) and contained a viscous brown fluid; the findings indicated a multifocal cystic lesion with septate walls (Figure 3b). Compositional testing of the liquid was not performed. Histologically, the cyst was covered by two layers of muscle covered by the chorioepithelial columnar epithelium (Figure 3c,d). Thereafter, no recurrence was observed after 2 years.

## DISCUSSION

Esophageal cysts are rare congenital malformations that were first described by Blassium in 1711.^1^ Esophageal cysts are embryologically derived from the foregut that result from defects in the tubulogenesis of the esophagus and are commonly found on the right side of the thoracic esophagus.^2^

Histologically, duplication cysts are located within the esophageal wall and have two layers of muscularis propria. They contain squamous or esophageal epithelium (columnar, cuboidal, and hairline epithelium).^3^ In this case, the chorioepithelial columnar epithelium covered the surface of the two smooth muscle layers that had reached the esophagus, and an esophageal duplication cyst was diagnosed.

The symptoms of esophageal duplication cysts vary. Esophageal duplication cysts in childhood may present with respiratory symptoms due to airway compression and stunted growth of the respiratory tissue. In adults, they may be asymptomatic and discovered incidentally or detected by bleeding, abscess formation, or dyspnea. Although rare, there have also been reports of cancer complications.^4^ Early surgical resection is desirable because the cysts do not regress spontaneously during childhood.

However, the disease may not increase in severity, even with follow‐up observation, and no treatment has been established for asymptomatic patients. Recently, endoscopic fenestrated resection using EUS has been reported.^5^ However, there have been reports of carcinogenesis,^4^ and the choice of treatment should be made with caution. In our case, the patient was asymptomatic, but a tendency toward enlargement was suspected due to the lack of previous indications. Considering the risk of future rupture, we opted for surgery, and no recurrence was observed.

Various modalities, including computed tomography, MRI, and EUS, are used to diagnose esophageal duplication cysts. In a typical case, MRI shows a low signal at T1, a high signal at T2, and an anechoic lesion on EUS.^6^ Location of the lesion within the esophageal wall and no evidence of internal cartilage are factors that distinguish an esophageal duplication cyst from a bronchogenic cyst. EUS allows direct observation and is thus useful for the diagnosis of esophageal duplication cysts.

However, the internal components of the cyst with fluid can be observed in various ways, depending on the degree of hemorrhage, protein, infection, and other components of the fluid; this can make diagnosis difficult. In our case, the lesion appeared to be contiguous with the muscularis propria, and we suspected an esophageal submucosal tumor. EUS fine‐needle aspiration (EUS‐FNA) has been recently reported for diagnosis; however, there have been reports of emergency surgery for infection after aspiration.^7^ Ali et al. reported that EUS‐FNA was performed in seven cases of hypoechoic lesions with suspected duplication cysts, and three of them were neoplastic lesions.^8^ Therefore, EUS‐FNA should be performed with caution.

CH‐EUS was performed in the present case. CH‐EUS provides a clear contrast with the surrounding parenchyma through stained images and microvessels continuously through the lesion. The contrast agent perflubutane can be used in patients with renal dysfunction, except for those with an egg allergy, and no serious side effects have been reported. CH‐EUS has been reported to be effective in differentiating mucus masses and nodules in intraductal mucinous neoplasms^9^; in the gastrointestinal tract, it is reported to be effective in distinguishing submucosal tumors, gastrointestinal stromal tumors, and leiomyomas.^10^ In our case, EUS showed that the lesion was filled with a viscous brown fluid that appeared hypoechoic; this suggested an esophageal submucosal tumor. Therefore, EUS‐FNA was also considered. However, CH‐EUS showed no blood flow inside because of the viscous fluid component, and there were no abnormal nodules in the lumen, which was observed as an anechoic area. Accordingly, a diagnosis of a benign esophageal duplication cyst was established. CH‐EUS was used in a clinical trial, and CH‐EUS appeared to be effective in diagnosing and differentiating esophageal duplication cysts.

Esophageal cysts present with various imaging findings. In Japan, CH‐EUS is covered by insurance for the liver and breast, and other areas are subject to clinical research. However, the efficacy in biliopancreatic^9^ and gastric^10^ lesions has been reported. In our case, CH‐EUS was effective in diagnosing esophageal duplication cysts. CH‐EUS should be used in a variety of cases in the future to study its effectiveness.

## CONFLICT OF INTEREST STATEMENT

None.

## ETHICS STATEMENT

CH‐EUS was performed at Aichi Medical University School of Medicine, approved by the institution's ethics committee (approval number 2015‐H209), and conformed to the code of ethics stated in the Declaration of Helsinki. The patient provided written informed consent.
